# Distribution of ε-Poly-l-Lysine Synthetases in Coryneform Bacteria Isolated from Cheese and Human Skin

**DOI:** 10.1128/AEM.01841-20

**Published:** 2021-04-27

**Authors:** Xinglin Jiang, Yulia Radko, Tetiana Gren, Emilia Palazzotto, Tue Sparholt Jørgensen, Tao Cheng, Mo Xian, Tilmann Weber, Sang Yup Lee

**Affiliations:** a The Novo Nordisk Foundation Center for Biosustainability, Technical University of Denmark, Kongens Lyngby, Denmark; b Qingdao Institute of Bioenergy and Bioprocess Technology, Chinese Academy of Sciences, Qingdao, People’s Republic of China; c Metabolic and Biomolecular Engineering National Research Laboratory, Department of Chemical and Biomolecular Engineering (BK21 Plus Program), Center for Systems and Synthetic Biotechnology, Institute for the BioCentury, Korea Advanced Institute of Science and Technology (KAIST), Daejeon, Republic of Korea; Norwegian University of Life Sciences

**Keywords:** ε-poly-l-lysine, NRPS, cheese bacteria, skin bacteria, antimicrobial, food preservative, epsilon-poly-l-lysine, epsilon-polylysine, polylysine

## Abstract

ε-Poly-l-lysine is a potent antimicrobial produced through fermentation of *Streptomyces* and used in many Asian countries as a food preservative. It is synthesized and excreted by a special nonribosomal peptide synthetase (NRPS)-like enzyme called Pls. In this study, we discovered a gene from cheese bacterium Corynebacterium variabile that showed high similarity to the Pls from *Streptomyces* in terms of domain architecture and gene context. By cloning it into Streptomyces coelicolor with a Streptomyces albulus Pls promoter, we confirmed that its product is indeed ε-poly-l-lysine. A comprehensive sequence analysis suggested that Pls genes are widely spread among coryneform actinobacteria isolated from cheese and human skin; 14 out of 15 *Brevibacterium* isolates and 10 out of 12 *Corynebacterium* isolates contain it in their genomes. This finding raises the possibility that ε-poly-l-lysine as a bioactive secondary metabolite might be produced and play a role in the cheese and skin ecosystems.

**IMPORTANCE** Every year, microbial contamination causes billions of tons of food wasted and millions of cases of illness. ε-Poly-l-lysine has potent, wide-spectrum inhibitory activity and is heat stable and biodegradable. It has been approved for food preservation by an increasing number of countries. ε-Poly-l-lysine is produced from soil bacteria of the genus *Streptomyces*, also producers of various antibiotic drugs and toxins and not considered to be a naturally occurring food component. The frequent finding of *pls* in cheese and skin bacteria suggests that ε-poly-l-lysine may naturally exist in cheese and on our skin, and ε-poly-l-lysine producers are not limited to filamentous actinobacteria.

## Abstract (precis)

Every year, microbial contamination causes billions of tons of food wasted and millions of cases of illness. ε-Poly-l-lysine has potent, wide-spectrum inhibitory activity and is heat stable and biodegradable. It has been approved for food preservation by an increasing number of countries. ε-Poly-l-lysine is produced from soil bacteria of the genus *Streptomyces*, also producers of various antibiotic drugs and toxins and not considered to be a naturally occurring food component.

## INTRODUCTION

The small cationic isopeptide ε-poly-l-lysine (ε-PL) is made from the essential amino acid l-lysine. It exhibits antimicrobial activity against a wide spectrum of bacteria, yeasts, and fungi by targeting the cell membrane and is heat stable and active in different food matrices (1). ε-PL has been a broadly used food preservative in Japan since the late 1980s, followed by Korea and China, and has been given generally-regarded-as-safe (GRAS) status in the United States, with U.S. GRAS number GRN000135. As a secondary metabolite, ε-PL was first isolated from the soil bacterium Streptomyces albulus, which is still used in its commercial production (2). Later, more producing strains were identified from the family of *Streptomycetaceae*, including the genera *Streptomyces* and *Kitasatospora*, and ergot fungi (3, 4). ε-PL is synthesized and excreted by a cell membrane-bound nonribosomal peptide synthetase (NRPS)-like enzyme named ε-PL synthetase (Pls). The structure and mechanism of *Streptomyces* Pls have been well studied (5). Biosynthetic regulation and the natural role of this compound are less well understood. Cheese prepared by fermentation of milk is an ancient food with a history of at least 8,000 years (6). The microorganisms on and in cheese and their secondary metabolites play key roles for the quality, preservation, safety, and flavor of the final cheese products. In this study, we discovered a Pls from cheese-isolated bacteria, confirmed its activity by heterologous expression, and investigated its distribution.

## RESULTS

**FIG 1:**
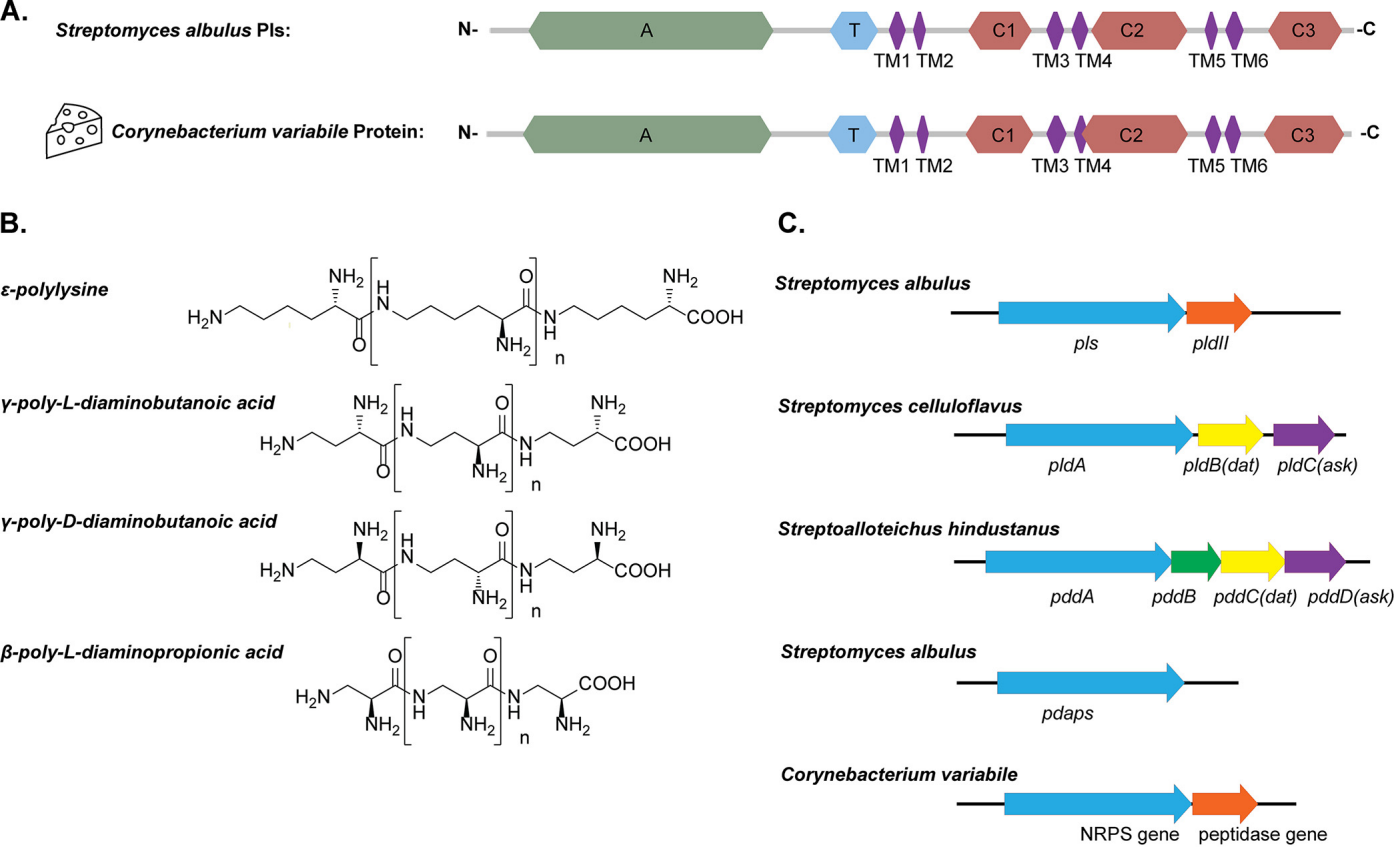
ε-PL synthetase from Corynebacterium variabile. (A) Comparison of protein domains between Pls from Streptomyces albulus and its homolog in Corynebacterium variabile. Domain analysis was performed by InterProScan (10). A, adenylation domain; T, thiolation domain; C1, C2, and C3, three C-terminal tandem domains related to acetyltransferases; TM1 to TM6, six transmembrane domains. (B) Chemical structures of the four known cationic homo poly-amino acids discovered in nature. (C) Gene contexts of the synthetases. Arrows of the same color indicate homologous genes. Genes are as follows: *pls*, ε-polylysine synthetase (17); *pldII*, ε-PL-degrading enzyme II (17); *pldA*, γ-poly-l-diaminobutanoic acid synthetase; *dat*, diaminobutyrate-2-oxoglutarate transaminase; *ask*, aspartate kinase (14); *pddA*, γ-poly-d-diaminobutanoic acid synthetase (14); and *pdabs*, β-poly-l-diaminopropionic acid synthetase (15).

When analyzing the genomes of cheese-isolated Corynebacterium variabile (7, 8) with antiSMASH (9), in all three sequenced strains, DSM 44702, Mu292, and NBRC 15286, we noticed a gene encoding an NRPS-like enzyme with high similarity to *Streptomyces* Pls. InterProScan (10) results showed that they share a unique domain architecture, which is not seen in typical NRPSs as found in the biosynthetic pathways of many peptide antibiotics, such as penicillin and vancomycin (11). The enzyme has a typical NRPS adenylation domain (A-domain) for substrate activation and a thiolation/peptidyl carrier protein domain for tethering the activated substrate. It does not have, however, the condensation domains or thioesterase domains typical of NRPSs. Instead, there are three tandem domains (C1, C2, and C3) related to acetyltransferases and six transmembrane (TM1 to TM6) domains separating the C1, C2, and C3 domains (Fig. 1A). Similar architectures can be found only in Pls-related β-poly-l-diaminopropionic acid (β-PDAP) synthetase, γ-poly-l-diaminobutanoic acid (γ-PLDAB) synthetase, and γ-poly-d-diaminobutanoic acid (γ-PDDAB) synthetase (12–14). β-PDAP, γ-PLDAB, and γ-PDDAB are cationic isopeptides structurally similar to ε-PL (Fig. 1B). β-PDAP and γ-PLDAB are coproduced with ε-PL in different *Streptomyces* strains, with higher antifungal activities and lower antibacterial activities than ε-PL (12, 13, 15). γ-PDDAB is produced by Streptoalloteichus hindustanus with strong antiviral activity and only weak antibacterial activities (14, 16). The *Corynebacterium* protein is more similar to Pls (sequence identity of 51%) than to the other three synthetases (32%, 33%, and 31%, respectively), and similar to the *Streptomyces* Pls gene, the *Corynebacterium* gene forms an operon with a peptidase gene, which is a different gene context than for the other three synthetases (Fig. 1C). The *Streptomyces* peptidase (PldII) was shown to be a ε-PL-degrading enzyme and postulated to have a self-protection function (17).

We tested ε-PL production from *C. variabile* using a two-stage culture method which was efficient in finding *Streptomyces* producers (18). However, no ε-PL was detected in the culture. We reasoned that the cheese bacteria may have different regulation of ε-PL biosynthesis from that of soil bacteria of the genus *Streptomyces*. Therefore, we cloned the *C. variabile* gene onto a plasmid with an inducible pBAD promoter. The recombinant plasmid was transferred into model organism Corynebacterium glutamicum. However, again ε-PL production could not be observed in the cultures with or without arabinose induction. We checked the enzyme expression by whole-cell proteomic analysis. The Pls expression in the recombinant strain after induction was confirmed, with an 8-fold increase of normalized signal abundance over the uninduced sample (see Data Set S1 in the supplemental material). In the sample of *C. variabile*, the signal of Pls protein was not detected, while 1,647 of the 2,972 predicted proteins were detected (Data Set S2).

**FIG 2:**
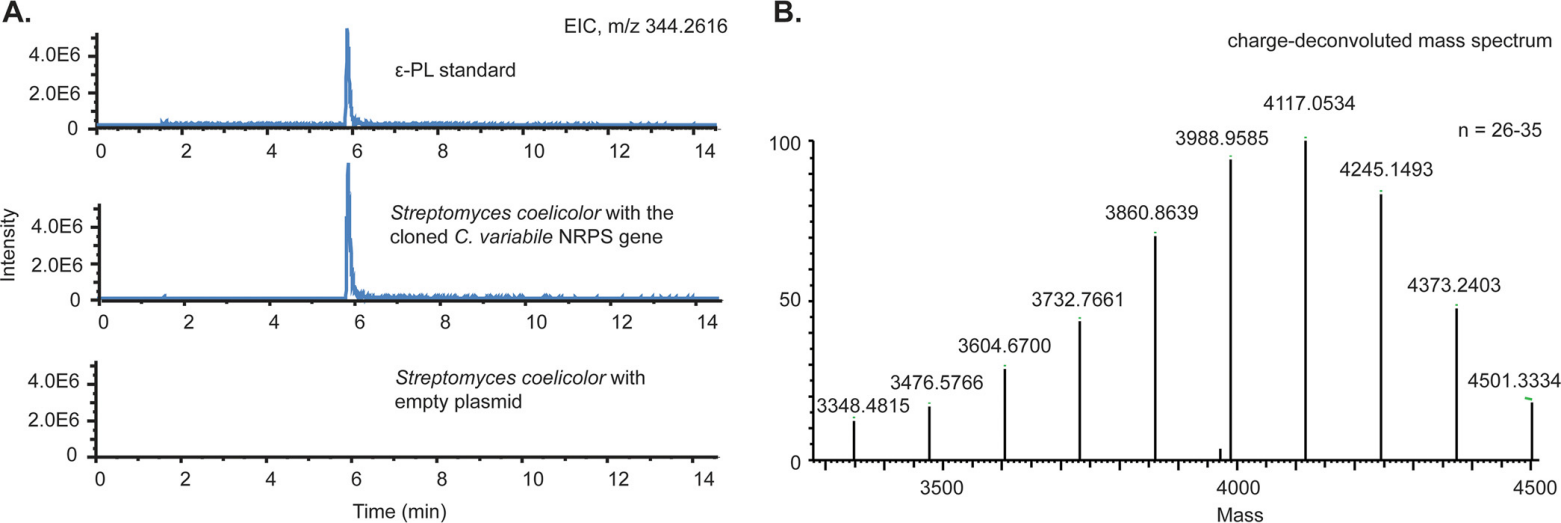
ε-PL production by heterologous expression of the *C. variabile* gene in Streptomyces coelicolor. (A) Bacterial culture extracts were analyzed by UHPLC-MS analysis. S. coelicolor with empty plasmid was used as a negative control. (B) Precise MS of the product match with ε-PL of 26 to 35 residues.

In *Streptomyces*, the promoter sequence is critical for ε-PL production. It has been demonstrated that expression of *pls* in the native host *S. albulus* with an altered promoter did not lead to ε-PL production, but the use of the original promoter resulted in ε-PL production even in a heterologous *Streptomyces* host (19). Inspired by this, we cloned the *C. variabile* gene under the control of the *S. albulus pls* promoter and transferred the plasmid into Streptomyces coelicolor M145, which does not have an endogenous *pls* gene. Using this expression system, ε-PL production was confirmed by ultrahigh-performance liquid chromatography (UHPLC) and high-resolution mass spectrometry (MS) (Fig. 2). The titer in shaking flask cultures was determined to be 120 ± 28 mg/liter by methylene blue agar diffusion assay (20).

**FIG 3:**
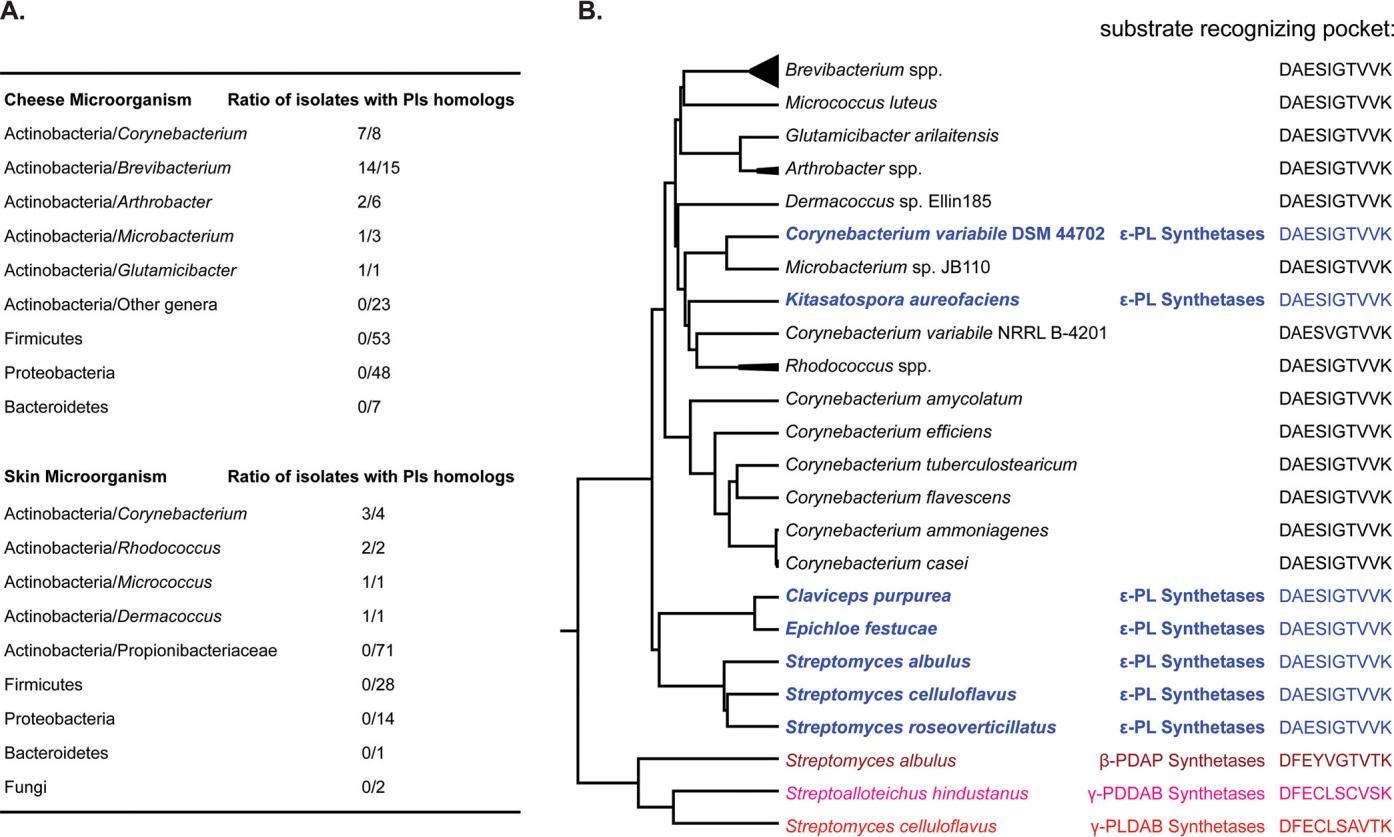
Pls distribution in microorganisms isolated from cheese and human skin. (A) Ratio of isolates with Pls homologs in different phyla and genera. The homologs were identified by BLASTP with a cutoff of 40% sequence identity and 80% sequence coverage. Detailed BLASTP results and the sequences of the homologs are listed in Data Set S3. (B) Protein phylogenetic tree and A-domain substrate-recognizing pocket according to Stachelhaus et al. (25). Experimentally confirmed small isopeptide synthetases and their hosts are shown in colors. Their protein sequence accession numbers are listed in Materials and Methods. The homologs from cheese and skin bacteria genomes are shown in black. Detailed NRPSpredictor2 results are included in Data Set S3.

We investigated the distribution of *pls* in a genome collection of 156 bacteria isolated from cheeses from Europe and the United States (21). As many microorganisms from surface-ripened cheeses can also be found in animal and human skin microbiota (22) and ε-PL is also used in cosmetic products (1), we further included a genome collection of 124 microorganisms isolated from human skin (23). We used experimentally confirmed Pls protein sequences as queries to do BLASTP against the two collections with cutoffs of 40% sequence identity and 80% sequence coverage. Pls homologs were found to be concentrated in coryneform actinobacteria, including *Corynebacterium*, *Brevibacterium*, *Arthrobacter*, *Microbacterium*, *Glutamicibacter*, *Rhodococcus*, *Micrococcus*, and *Dermacoccus*. No hit was found in bacteria from other genera or phyla (Fig. 3A). No hit was found using the other three synthetases as queries with the same cutoffs.

Phylogenetic analysis shows that all the hits cluster together with the experimentally confirmed Pls from *C. variabile*, *Kitasatospora*, *Streptomyces*, and fungi, while the synthetases of the other three isopeptides are on more distant branches (Fig. 3B). Most importantly, NRPSpredictor2 (24) results show that the 10-residue substrate-recognizing pockets (25) of the coryneform bacterial proteins are identical or highly similar to that of the confirmed Pls proteins but substantially different from those of the other three synthetases (Fig. 3B), which strongly suggests that their substrate is lysine.

## DISCUSSION

In this study, we confirmed that the cheese bacterium *C. variabile* DSM 44702 harbors a functional ε-PL synthetase gene. However, we did not observe ε-PL production by *C. variabile* under our artificial culturing conditions. This is likely caused by a regulatory mechanism that requires an unknown trigger signal, which was missing in our cultivations. Such tightly controlled biosynthetic pathways are very common in microbial secondary metabolite biosynthesis and thus have shaped the term “silent biosynthetic gene cluster” (26). Corynebacteria are related to *Streptomyces* and have many properties desired for industrial fermentation, like being nonfilamentous and having faster growth, a simpler life cycle, and a simpler secondary metabolism. Successful activation of ε-PL synthesis in corynebacteria may provide the basis for a new ε-PL production process. Furthermore, Pls were widely found in cheese- and skin-isolated coryneform bacteria. The majority of *Brevibacterium* and *Corynebacterium* isolates, which are among the most important microorganisms in cheese production and also commonly found on human skin, have Pls. It is possible that ε-PL naturally exists in cheese and on human skin and may have a role in their ecologies. Other antimicrobial compounds, like bacteriocins, have been known to be produced in cheese and skin environments and modulate the microbiota compositions (27, 28). The existence and quantity of ε-PL on human skin and in different cheeses and different stages of the cheese making process require further study.

## MATERIALS AND METHODS

### Bacteria.

Corynebacterium variabile DSM 44702 was obtained from the DSMZ. Streptomyces coelicolor M145 and Corynebacterium glutamicum MB001(DE3) were used as the heterologous hosts. Escherichia coli DH5α was used for DNA cloning.

### Gene cloning.

An expression vector pXJ0GC for corynebacteria was developed from shuttle plasmid pAL374 (29). An AraC-pBAD fragment was amplified from pBAD18 with primers xj336.1 and xj337. An rrnBT1T2 fragment was amplified from pBAD30 with primers xj338 and xj339. An *aac(3)-oriT* fragment was amplified from pRM4.3 with primers xj340 and xj341. A replication origin fragment was amplified from pAL374 with primers xj342 and xj343. An mScarlet-FDterminator fragment was chemically synthesized. The above-mentioned fragments were assembled by Gibson reaction into plasmid pXJ00. A pT7-pTrc-gfp-cmr fragment was amplified from pACY-gfp with primers julie11 and julie12. It was assembled with pXJ100 digested with SfaAI, HindIII, and NdeI, resulting in plasmid pXJ0GC. The *C. variabile* Pls gene was PCR amplified from *C. variabile* DSM 44702 genomic DNA with primers xj372 and xj373 and cloned onto the vector backbone amplified from pXJ0GC with primers bb0s and bb0a. The resulting plasmid, pXJ146, was used for gene expression in C. glutamicum. The promoter sequence of the *S. albulus* Pls gene was chemically synthesized and then amplified with primers xj420 and xj421. The *C. variabile* Pls gene was PCR amplified from *C. variabile* DSM 44702 genomic DNA with primers xj426 and xj427. A plasmid backbone was amplified from shuttle vector pRM4e with primers xj422 and xj423. The above-mentioned three fragments were assembled by Gibson reaction into plasmid pXJ155CV and used for gene expression in S. coelicolor. Primer sequences and DNA sequences chemically synthesized are listed in Tables 1 and 2.

**TABLE 1:** Primer oligonucleotides used in this study

Primer	Sequence (5′–3′)
xj336.1	GACCTCCTCAATTCGCTAGCCCAAAAAAACGGGTATGGAGAAACAG
xj337	CTACAAACTCTTTAATTAAGCATAATGTGCCTGTCAAATGGACG
xj338	GCACATTATGCTTAATTAAAGAGTTTGTAGAAACGCAAAAAGG
xj339	CCCTTTTGCTGATGGAGCTGCACATGAACCGGCTGTTTTGGCGGATGAGAG
xj340	GGTTCATGTGCAGCTCCATCAGC
xj341	AATTCGCCCTTGCTCCCGGGCAGGATAGGTGAAGTAGG
xj342	ACCTATCCTGCCCGGGAGCAAGGGCGAATTGTAACCG
xj343	GGTGAGGTTATGGCGGAGGGTT
julie11	TGGGCTAGCGAATTGAGGAGGTCTAGCGATCGCACGCTCTCCCTTATGCGACTCC
julie12	GAGCCTTTAATTGTATCGGTTTAGCGATTTCGCAGTCGAACGACCGAGC
xj372	TGGGCTAGCGAATTGAGGAGGTCTAGCGATGTGCTTGACTGACGTCGTGAAACTC
xj373	CGCAGTCGAACGACCGAGCGTAGCGAGTCCGGACCGATCCTATGTCGTTGACTG
bb0s	GACTCGCTACGCTCGGTCGTTCG
bb0a	ATCGCTAGACCTCCTCAATTCGCTAGCC
xj426	GGCACCGAACAGAGGCATATCGATGTGCTTGACTGACGTCGTGAAACTCACC
xj427	TGTGGATAACCGTATTACCGCCTCACGATCCTATGTCGTTGACTGGTAGGCG
xj422	GGTGCGGCCGCCTGAGGCGGTAATACGGTTATCCACAGAATCAG
xj423	GGATGTTCACGGCCCGTTGCGCTCACTGCCCGCTTTC
xj420	GGCAGTGAGCGCAACGGGCCGTGAACATCCTCAAGTAGGC
xj421	CATCGATATGCCTCTGTTCGGTGCCG

**TABLE 2:** DNA fragments chemically synthesized

DNA fragment	Sequence
mScarlet-FDterminator	AAGAAAACCTTGAGGGGCAGGGCAGCTTATATGCTTCAAAGCATGACTTCCTCTGTTCTCCTAGACCTCGCAACCCTCCGCCATAACCTCACCACGTTGAAAATCTCCAAAAAAAAAGGCTCCAAAAGGAGCCTTTAATTGTATCGGTTTAGCGATTTAAATTTACTTGTACAGCTCATCCATGCCTCCCGTCGAATGGCGGCCTTCTGAACGTTCATATTGTTCTACCACAGTGTAATCCTCATTATGGCTGGTGATGTCCAATTTGCGATCCACGTTGTAAGCACCCGGCATTTGAACGGGCTTTTTCGCCTTGTAAGTCGTTTTGAAGTCGGCCAAATAGCGACCTCCGTCTTTAAGACGCAGAGCCATTTTAATGTCGCCCTTCAGGACACCGTCTTCGGGATACAGGCGTTCCGTCGATGCCTCCCAGCCCATCGTTTTCTTTTGCATCACCGGACCATCGGGTGGGAAGTTAGTGCCACGCAATTTGACTTTGTAAATGAGAGTACCATCCTCCAGGGAAGTATCTTGGGTAACAGTTACGGCACCTCCGTCTTCGAAGTTCATCACCCGCTCCCACTTAAAACCCTCCGGGAAGGACTGTTTATAGTAGTCTGGAATGTCTGCTGGGTGTTTCGTGAAAGCACGGCTTCCGTACATGAACTGTGGAGACAAAATGTCCCAGCTAAATGGCAGGGGGCCACCCTTGGTTACTTTCAGTTTAGCAGTCTGAGTACCCTCATAAGGACGACCTTCTCCTTCACCCTCGATCTCGAACTCGTGACCATTCATGCTTCCTTCCATGTGTACCTTAAACCGCATAAACTCCTTGATCACTGCTTCTCCTTTGCTGACCATTATAATTTCCTGTGTGAAATTGTTATCCGCTCACAATTCCACACAACATACGAGCCGGAAGCATAAAGTGTAAACGCGATCGCTAGACCTCCTCAATTCGCTAGCCCAAAAAAACGGGTATGGAGAAACAGTAGAGAGTTGCGATAAAAAGCGTCAGGTAGGATCCGCTAATCTTATGGATAAAAATGCTATGGCATAGCAAAGTGTGACGCCGTGCAAATAATCAATGTGGACTTTTCTGCCGTGATTATAGACACTTTTGTTACGCGTTTTTGTCATGGCTTTGGTCCCGCTTTGTTACA
*S. albulus pls* promoter	GGGCCGTGAACATCCTCAAGTAGGCGGCGCCGGCGCCCCGTTGGTCGACGTCCGCGGGCGTCTCGGGGTGTACCGGACGCACGGCGAGGGTATGGCCGTGTCATGACACCGCGATGAGGTCGGCGTGAGAAGTCGATGAATATGTGCTCAGTTGCGCAATCATTTAGACAAGGCTTGACCGGTTGACCCGTGACCGATCGGGATCACGGTCCTGACCTGCGGTTTTATCGGCACGGGGGAGTGGTGCCGAAAACAATCCCCGGCCCGAGTCAATTCTTTCCCACGCCGTGGTCAGGCGCCGCGGCCGCTTTCCCGGCGCCGCCTGCCCAAGCGCCCGATGGCCGCTTTCACAGCACGTTCGAATTGCGGAACAGACCGCGCGGCAGGCGAACCCGCTGCCTGAGCAGCGACATCTCTAGGGGCGAACGTCCGAGGGTCATCCACCCACCGGCACCGAACAGAGGCATATCGATGTGCTTGACTGACGTCGTGAAACTCAC

### Culture conditions.

*Streptomyces* and *Corynebacterium* strains were maintained on ISP2 agar (BD Difco). They were assayed for ε-PL production by a two-stage cultivation method (18). S. coelicolor strains were inoculated in M3G medium (30) at pH 6.8 for 24 h at 30°C, and then the pH was adjusted to 4.0 by HCl and culture was continued for another 3 days with shaking at 120 rpm. *Corynebacterium* strains were cultured similarly with GMPY medium (malt extract at 10 g/liter, peptone at 10 g/liter, and yeast extract at 0.1 g/liter, autoclaved, with glucose added at 10 g/liter as a carbon source), and 1% arabinose was used for induction of gene expression in recombinant strains.

### Extraction.

A Bond Elut LRC-CBA column (Agilent; part number 12113037) was conditioned by washing with 5 ml of methanol and then 5 ml of water. Bacterial culture supernatant was adjusted to pH 8 with NaOH and loaded on the column with a speed of 3 ml per min. The column was washed with 5 ml of water and then eluted with 5 ml of methanol twice. The elution was dried in a rotary evaporator at 38°C, redissolved and collected with 1 ml of methanol, and then concentrated to 50 μl using a vacuum centrifuge.

### UHPLC-MS analysis.

UHPLC-MS analysis of the extract was performed on a Dionex Ultimate 3000 UHPLC system coupled to a high-resolution Orbitrap Fusion mass spectrometer (Thermo Fisher Scientific, Waltham, MA) and a UV-visible (UV/Vis) diode array detector (DAD). Separate positive- and negative-ion-mode electrospray ionization (ESI) experiments were carried out with an MS scan range of 100 to 1,000 Da. Injections of 8 μl of each sample were separated using a Waters Cortecs T3 column, 150 by 2.1 mm (inside diameter [i.d.]) and 1.6-μm particle size, at a temperature of 35.0°C and a flow rate of 0.35 ml/min. Elution was performed with 0.1% formic acid in water (mobile phase A) and 0.1% formic acid in acetonitrile (mobile phase B) in a multistep program: 0% mobile phase B for 2.5 min, a linear gradient from 0% to 100% mobile phase B in 15 min, 100% mobile phase B for 2 min, and 0% mobile phase B for 2 min.

### Methylene blue agar diffusion assay.

The ε-PL titer of the *Streptomyces* culture was determined by methylene blue agar diffusion assay as described in a previous paper (20). The agar plates were made with 0.75% agar and 0.002% methylene blue. A 100-μl sample was applied to the plate and incubated at 30°C for 5 h before the diffusion diameter was measured. A standard curve was made from six ε-PL concentrations ranging from 50 to 1,000 mg/liter. A regression coefficient of 0.9948 was achieved.

### Proteomic analysis.

Cells were collected by centrifugation at 12,000 × *g* for 10 min 48 h after the inoculation and stored at −20°C until analyzed. After thawing of the cells on ice, the samples were centrifuged again and any remaining supernatant was removed. The samples were added with two 3-mm zirconium oxide beads (Glen Mills, Clifton, NJ) and then moved away from ice and immediately added with 100 μl of 95°C guanidinium HCl solution [6 M guanidinium hydrochloride, 5 mM tris(2-carboxyethyl)phosphine, 10 mM chloroacetamide, and 100 mM Tris-HCl (pH 8.5)]. Cell disruption was performed in a mixer mill (MM 400; Retsch, Haan, Germany) set at 25 Hz for 5 min at room temperature, followed by 10 min in a ThermoMixer at 95°C at 2,000 rpm. Remaining cell debris was precipitated by centrifugation at 15,000 × *g* for 10 min. A 50-μl volume of the supernatant was collected and diluted with 50 μl of 50 mM ammonium bicarbonate. The protein concentration was determined by bicinchoninic acid (BCA) assay; 100 μg of protein was subjected to tryptic digestion at constant shaking (400 rpm) for 8 h and then added with 10 μl of 10% trifluoroacetic acid (TFA). The samples were cleaned by stage tipping using C_18_ resin (Empore; 3M, USA).

The proteomic analysis was carried out on a CapLC system (Thermo Scientific) coupled to an Orbitrap Q Exactive HF-X mass spectrometer (Thermo Scientific). Samples was first injected and carried at a flow rate of 10 μl/min on a precolumn (μ-precolumn C_18_ PepMap 100, 5 μm, 100 Å) and then at a flow of 1.2 μl/min on a 15-cm C_18_ EASY-Spray column (PepMap RSLC C_18_, 2 μm, 100 Å, 150 μm by 15 cm) for peptide separation. The mobile phase gradient increased from 4% to 76% acetonitrile in water over a total of 60 min. The mass spectrometer was operated in data-dependent mode with Orbitrap resolution set to 60,000 and the following parameters: AGC target, 3.0e6; maximum injection time, 50 ms; intensity threshold, 5.0e3; and dynamic exclusion, 25 s. Data-dependent MS2 selection was carried out in top 20 speed mode with high-energy collisional dissociation (HCD) with collision energy set to 28% (AGC target, 1.0e4; maximum injection time, 22 ms; isolation window, 1.2 *m/z*).

Proteome Discoverer 2.3 was used for analysis of the Thermo raw files with the following settings: fixed modifications, carbamidomethyl (C), and variable modifications, oxidation of methionine residues. First-search mass tolerance was 20 ppm, and tandem MS (MS/MS) tolerance was 20 ppm. Trypsin was used as the digestion enzyme, and one missed cleavage was allowed. The false-discovery rate (FDR) was set at 0.1%. The match-between-runs window was set to 0.7 min. Quantification was based only on unique peptides, and normalization between samples was based on total peptide amount. For the searches, a protein database consisting of the reference proteome in combination with the expressed target proteins was used.

### Bioinformatics.

The genome sequences used in antiSMASH analysis were downloaded from NCBI with accession numbers NC_015859.1 for *C. variabile* DSM 44702, GCA_900015285.1 for *C. variabile* Mu292, and GCA_006539825.1 for *C. variabile* NBRC 15286. The sequences of experimentally confirmed Pls and related synthetases were downloaded from NCBI as Epichloë festucae ε-PL synthetase (accession number BBU42014.1) (4), Claviceps purpurea ε-PL synthetase (accession number CCE28893.1) (4), *S. albulus* NBRC14147 ε-PL synthetase (accession number BAG68864.1) (5), Kitasatospora aureofaciens ε-PL synthetase (accession number AZL89021.1) (31), *S. celluloflavus* ε-PL synthetase (NCBI GenPept accession number WP_110952033.1) (13), Streptomyces roseoverticillatus MN-10 ε-PL synthetase (NCBI GenPept accession number BAH85292.1) (32), *C. variabile* DSM 44702 ε-PL synthetase (NCBI GenPept accession number WP_041630296.1), *S. albulus* strain NBRC 14147 β-PDAP synthetase (NCBI GenPept accession number EXU85975.1) (12), *S. celluloflavus* γ-PLDAB synthetase (NCBI GenPept accession number WP_110952768) (14), and S. hindustanus γ-PDDAB synthetase (NCBI GenPept accession number WP_083959783) (14).

Protein domain analysis was performed by InterProScan (10). Phylogenetic analysis was done by MEGA-X using Muscle for multiple-sequence alignment and Poisson model for UPGMA (unweighted pair group method using average linkages) tree building. NRPS A-domain substrate prediction was done by NRPSpredictor2 (24). For the cheese microorganism genome data set, 156 genomes were downloaded directly from the Data Set S1 file of reference 21, and the amino acid sequences were extracted from the GenBank format files using CLCgenomics (v.20.0). In addition to this, we found updated genomes for 47 of the 156 strains in NCBI. The genome accession numbers are listed in Data Set S3. Genes were downloaded from NCBI and included in the analysis. For the human skin microorganism genome data set from reference 23, 124 genomes were downloaded from the NIH Human Microbiome Project (https://www.hmpdacc.org/hmp/catalog/grid.php?dataset=genomic). We downloaded the following Pls proteins to use as a reference: those with NCBI GenPept accession numbers BAG68864.1, BAH85292.1, WP_041630296.1, AZL89021.1, CCE28893.1, and BBU42014.1. We then used BLASTP (v.2.6.0+) with the following parameter to identify putative Pls in the downloaded amino acid data sets: -evalue 0.000001. We then extracted hits with at least 40% identity and at least 80% coverage of the reference proteins.

## Supplementary Material

Supplemental file 1

Supplemental file 2

Supplemental file 3
